# Intravitreal drug injection for glaucoma: mechanisms, clinical efficacy, and future horizons

**DOI:** 10.3389/fphar.2025.1660401

**Published:** 2025-08-13

**Authors:** Bin Lin, Peng Shi, Dong-Kan Li

**Affiliations:** ^1^ Xiamen Eye Center and Eye Institute of Xiamen University, School of Medicine, Xiamen, China; ^2^ Xiamen Clinical Research Center for Eye Diseases, Xiamen, Fujian, China; ^3^ Xiamen Key Laboratory of Ophthalmology, Xiamen, Fujian, China; ^4^ Fujian Key Laboratory of Corneal & Ocular Surface Diseases, Xiamen, Fujian, China; ^5^ Xiamen Key Laboratory of Corneal & Ocular Surface Diseases, Xiamen, Fujian, China; ^6^ Translational Medicine Institute of Xiamen Eye Center of Xiamen University, Xiamen, Fujian, China

**Keywords:** intravitreal injection, glaucoma, anti-vegf, sustained-release technology, minimally invasive surgery

## Abstract

Intravitreal drug injection has emerged as a transformative approach in glaucoma management, overcoming the limitations of traditional treatments such as poor compliance with topical medications and high complication rates of filtration surgery. This review synthesizes the mechanisms, clinical efficacy, and future directions of intravitreal drug injection in glaucoma management, with a focus on Anti-vascular endothelial growth factor (anti-VEGF) agents, sustained-release preparations, and intraoperative adjuvant injections. Anti-VEGF drugs, as the cornerstone for neovascular glaucoma (NVG), effectively regress iris neovascularization and reduce intraocular pressure (IOP), with aflibercept achieving an 86.7% regression rate and a 12.3 mmHg IOP reduction in clinical trials. Sustained-release preparations, leveraging porous structures or biodegradable carriers with differential pore sizes or degradation rates, enable long-term drug release (up to 6 months) and stable 1OP control, addressing the need for frequent injections. Intraoperative adjuvant injections, such as epinephrine during minimally invasive glaucoma surgery (MIGS), further enhance surgical success by reducing scarring and improving IOP control. Despite these advancements, challenges remain, including reliance on primary disease control for anti-VEGF efficacy, carrier displacement risks, and the lack of real-time drug concentration monitoring. Emerging technologies, such as intelligent responsive delivery systems, nanorobotics, and Clustered Regularly Interspaced Short Palindromic Repeats and CRISPR-associated protein 9 (CRISPR-Cas9) gene editing, offer promising solutions to achieve precise, individualized therapy. This review highlights the shift from passive IOP reduction to active neurovascular regulation, emphasizing the potential of intravitreal injection to redefine glaucoma treatment paradigms.

## 1 Introduction: the positioning of intravitreal injection in glaucoma treatment

Glaucoma, the primary cause of irreversible blindness globally, is undergoing a paradigm shift in its treatment model from traditional methods to precise, minimally invasive techniques (Lin et al., 2024). Conventional treatments such as topical medication have limitations of poor compliance, with only 50% of patients able to adhere to medication, and filtration surgery has a high complication rate, with approximately 30% of cases developing shallow anterior chamber or filtration bleb scarring (Shah et al., 2021; Mercieca and Weber, 2025). Epidemiological data show that among the approximately 80 million glaucoma patients worldwide, 15%–20% are drug-refractory, and the incidence of neovascular glaucoma (NVG) in Asian populations accounts for 5.8% of all glaucoma types (Talaat et al., 2021; Massenzio et al., 2023). Intravitreal injection, which breaks through the blood-ocular barrier and directly delivers anti-Vascular Endothelial Growth Factor (anti-VEGF) drugs to target tissues, has shown unique advantages in the treatment of NVG. Combining anti-glaucoma surgery with intravitreal injection can increase the short-term success rate by 40% and significantly inhibit iris neovascularization (Zhou et al., 2023; Bai et al., 2021). Nanocarrier technology, which enables sustained release by loading neuroprotective drugs such as brimonidine nanoparticles, is expected to address the bottleneck of monthly repeated injections required for traditional intravitreal injections (Pei et al., 2024; Scheive et al., 2021).

In recent years, minimally invasive technologies have driven a shift in treatment strategies from passive intervention to active regulation. For instance, the Xen Gel Stent implant enables aqueous humor drainage through a small incision, reducing the number of medications used by 2.3 ± 1.1 types in Primary Open-Angle Glaucoma (POAG) patients 1 year after surgery (Yang et al., 2022; Wang C. et al., 2024). Technological innovations and the renewal of treatment concepts are reshaping the landscape of glaucoma management. As a new minimally invasive approach, ultrasonic cycloplasty (UCP) achieves a 1-year success rate of 68.4% in advanced refractory glaucoma, with a 60% lower complication rate compared to traditional cyclophotocoagulation (Longfang et al., 2022). A 2024 expert survey in Japan shows that 86% of surgeons have adopted Minimally Invasive Glaucoma Surgery (MIGS) combined with cataract surgery as the preferred option, reflecting that minimally invasive technologies have evolved from supplementary measures to first-line choices (Iwasa et al., 2025; Chan et al., 2023). These breakthroughs mark the entry of glaucoma treatment into the era of “precision intervention”, providing personalized solutions for patients at different stages through the integration of targeted drug delivery, minimally invasive surgery, and continuous monitoring technologies (Gillmann and Mansouri, 2020; Micheletti et al., 2024).

Notably, this review introduces a novel analytical framework: shifting from the traditional focus on passive intraocular pressure (IOP) lowering to active regulation of the neurovascular microenvironment. By integrating evidence from anti-VEGF-mediated neovascular regression, sustained-release drug delivery for long-term neuroprotection, and intraoperative adjuvant therapies for surgical optimization, we highlight how intravitreal injection acts as a “multi-target hub”-bridging pharmacology, surgical innovation, and molecular regulation. This holistic perspective distinguishes our work from fragmented summaries of individual therapies.

## 2 Mechanism of action: drug categories and neurovascular regulatory pathways

To elaborate on this multifaceted regulatory role, we first dissect the core mechanisms of intravitreal therapy, focusing on three key drug categories and their interactions with the eye’s neurovascular network.

### 2.1 Anti-VEGF drugs: core therapy for neovascular glaucoma

Anti-VEGF drugs have become the core treatment for NVG. Their pathological mechanism mainly stems from overexpression of Vascular Endothelial Growth Factor-A (VEGF-A) caused by retinal ischemic diseases such as diabetic retinopathy and retinal vein occlusion, which in turn leads to the formation of abnormal neovascularization in the iris and anterior chamber angle, ultimately resulting in angle adhesion, closure, and a sharp increase in intraocular pressure (Rittiphairoj et al., 2023). Studies have shown that intravitreal injection of anti-VEGF drugs can specifically block VEGF-A activity and effectively inhibit the proliferation of vascular endothelial cells. Clinical data indicate that iris neovascularization regresses in more than 85% of patients (Yun et al., 2025). Clinical trials have confirmed that anti-VEGF therapy can significantly reduce intraocular pressure with an average reduction of 7–15 mmHg, while reducing angle adhesion and creating favorable conditions for subsequent surgical treatment (Inatani et al., 2021). In terms of combined therapy, the combination of anti-VEGF drugs with Ahmed Glaucoma Valve Implantation (AGVI) or trabeculectomy can significantly improve surgical success rates. A meta-analysis shows that preoperative use of bevacizumab can increase the success rate of AGVI by 23% (Hwang and Lee, 2021), and postoperative intraocular pressure control is more stable (Lin et al., 2022). Another study confirms that compared with the surgery group, the treatment group receiving anti-VEGF combined with anti-glaucoma surgery has a 35% higher success rate at 6 months after surgery, with a significantly lower incidence of complications (Zhou et al., 2023; Guo et al., 2021). It is worth noting that there are differences in efficacy among different anti-VEGF drugs: a network meta-analysis indicates that aflibercept is superior to ranibizumab in reducing intraocular pressure with a mean difference of 2.1 mmHg, while conbercept performs better in neovascular regression rate with a relative risk of 1.15 (Wang J. et al., 2024). These findings provide an important basis for clinical individualized medication.

### 2.2 Sustained-release preparations: long-term intraocular pressure control and neuroprotection​

**FIGURE 1:**
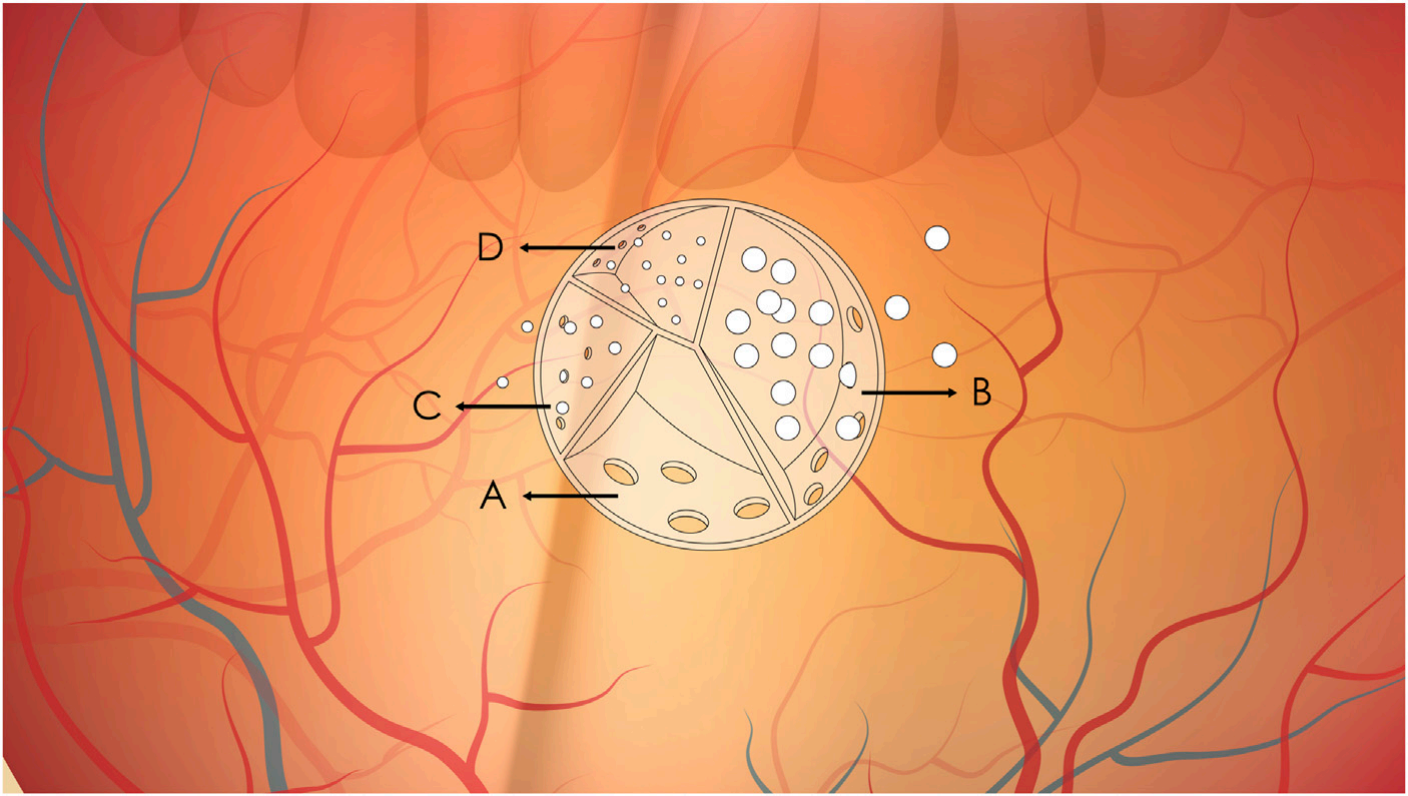
Schematic diagram of sustained drug release from intravitreal sustained-release carriers. **(A–D)** Represent compartments with sieve pores of decreasing size (A > B > C > **(D)**. **(A)** Complete drug release due to large pores; **(B)** Ongoing release with moderate pore size; **(C, D)** Minimal release due to small pores.

Current research has shown that sustained-release technologies can significantly extend the duration of drug action. Sustained-release drug carriers achieve controlled drug release through porous structures or biodegradable materials. As illustrated, the interior of the carrier is divided into multiple independent units, each regulating drug release rate via “sieve pores” (porosity) of different sizes or differentiated degradation rates. This is similar to a ‘layered medicine box’—compartments with larger pores (like wide-mouthed bottles) release drugs quickly at first, while those with smaller pores (like narrow-mouthed bottles) let drugs seep out slowly, ensuring a steady supply over months (So et al., 2025; Li et al., 2025). This design overcomes the drawback of frequent administration required for traditional intravitreal injections, enabling sustained drug concentrations from several weeks to months following a single injection (Long et al., 2024; Lee et al., 2023). As shown in Figure 1, drugs are encapsulated in a large “sustained-release carrier”, which can be hydrogel microspheres, biodegradable implants, or nanoparticles. The drug within the carrier is contained in different small compartments, each equipped with a sieve pore of varying sizes for drug release. The compartment with the largest sieve pore will have its drug released completely first, while the one with the smallest sieve pore allows the slowest drug release. This design ensures that the drug in the carrier can be continuously released into the eye over an extended period after injection, thereby achieving long-term and stable drug concentrations. For different disease conditions and different drugs, the number and size of compartments in the sustained-release carrier, as well as the quantity ratio and size ratio of sieve pores, can be designed accordingly.

For example, bimatoprost-containing hydrogel microsphere carriers can achieve sustained release for 3–6 months, while brinzolamide-containing biodegradable implants can maintain drug efficacy for more than 6 months. This long-acting release property not only avoids the risk of intraocular pressure fluctuations caused by frequent injections but also significantly improves patient compliance (Rodrigo et al., 2021; Ham et al., 2023). In terms of pharmacological mechanisms, prostaglandin analogs promote aqueous humor outflow through the uveoscleral pathway by upregulating matrix metalloproteinases, while carbonic anhydrase inhibitors reduce aqueous humor production by 30%–40% through inhibiting ciliary body carbonic anhydrase. These two mechanisms provide dual guarantees for intraocular pressure control (Soomsawasdi et al., 2025). It is worth noting that intravitreal injection of bone marrow mesenchymal stem cells (MSCs) has shown a protective effect on retinal ganglion cells in animal models of advanced glaucoma, opening up a new avenue for neuroprotective therapy in glaucoma (Vilela et al., 2021). Future development directions need to focus on combined treatment strategies and new delivery systems. Clinical evidence indicates that Adeno-Associated Virus (AAV) vector-mediated Apolipoprotein A-I Binding Protein (AIBP) gene therapy exhibits regulatory effects on retinal cholesterol metabolism and neuroprotective effects in experimental glaucoma (Ju et al., 2025). The latest research has also found that intravitreal injection of anti-High Mobility Group Box 1 (HMGB1) monoclonal antibody can effectively regulate inflammatory responses in animal models of glaucoma, providing a theoretical basis for the development of sustained-release preparations targeting neuroinflammation (Tonner et al., 2022). In terms of technical carriers, the optimized design of hydrogel microspheres and biodegradable implants will further improve the sustained-release performance and targeting of drugs in the vitreous cavity (Prajapati et al., 2021).

### 2.3 Anti-fibrotic/anti-inflammatory drugs: surgical adjuvant therapy​

The occurrence and development of glaucoma are closely related to intraocular inflammatory changes (Lin and Li, 2025). In terms of anti-fibrotic and anti-inflammatory adjuvant therapy, studies have shown that 0.01% epinephrine can significantly inhibit trabecular meshwork cell contraction by down-regulating the expression of Actin Alpha 2 (ACTA2) gene, and this mechanism can reduce the risk of filtration tract scarring by 50% (Li et al., 2023). Meanwhile, lipid nanoparticle delivery systems targeting the Myocardin-Related Transcription Factor/Serum Response Factor (MRTF/SRF) pathway have shown specific inhibitory effects on trabecular meshwork fibrosis (Luo et al., 2022), and some natural extracts have also been confirmed to antagonize Transforming Growth Factor Beta 2 (TGF-β2)-induced trabecular meshwork cell fibrosis (Wu et al., 2024). Sirtuin 1 (Sirt1) activators exert effects through dual mechanisms: they both inhibit oxidative stress and block Transforming Growth Factor Beta-induced fibrotic signaling pathways (Tie et al., 2025). In the field of anti-inflammatory therapy, anti-Tumor Necrosis Factor Alpha (TNF-α) agents (such as adalimumab) alleviate optic nerve inflammatory damage by inhibiting microglial activation (Meng et al., 2023), which is consistent with the mechanism by which Sesamol regulates neuroinflammation through the AMP-Activated Protein Kinase/Sirtuin 1/Nuclear Factor Kappa-Light-Chain-Enhancer of Activated B Cells (AMPK/SIRT1/NF-κB) pathway (Feng et al., 2022). It is worth noting that small interfering RNA for Secreted Protein Acidic and Cysteine Rich (siSPARC) gene silencing technology combined with gelatin hydrogel has shown better anti-scarring effects than mitomycin C in animal models (Chun et al., 2021), providing a new option for surgical adjuvant therapy. In terms of target innovation, existing studies are breaking through the traditional anti-hypertensive framework. In addition to epinephrine, polyphenolic antioxidants have become potential therapeutic targets by protecting the trabecular meshwork from oxidative damage (Sacca et al., 2020), and Matrix Metalloproteinases (MMPs) modulators can simultaneously intervene in trabecular meshwork remodeling and optic nerve protection (Zhang et al., 2023). In the innovation of drug delivery systems, lipid nanoparticles (Luo et al., 2022) and positively charged, tuned gelatin hydrogels (Chun et al., 2021) have significantly improved the intraocular bioavailability of anti-fibrotic drugs. For pressure fluctuations caused by repeated intraocular injections, prophylactic use of anti-glaucoma drugs can reduce the peak intraocular pressure after injection by 35% (Soomsawasdi et al., 2025). These advances have jointly promoted the transformation of intraocular injection from a single treatment to a multi-modal treatment integrating anti-hypertension, anti-fibrosis, and neuroprotection. Among them, the combination strategy of anti- Tumor Necrosis Factor Alpha (anti-TNF-α) agents and anti-VEGF drugs has shown a synergistic effect in neovascular glaucoma (Zhou et al., 2023; Carrola et al., 2021).

A summary of the key mechanisms, efficacy outcomes, and supporting references for the drug categories discussed in this section is provided in Table 1.

**TABLE 1:** Mechanisms, key efficacy outcomes, and supporting references of intravitreal drugs for glaucoma.

Drug Category	Core Mechanism	Key Efficacy Outcomes	References
Anti-VEGF Drugs	Inhibit VEGF-A activity to block abnormal neovascularization in iris and anterior chamber angle	*86.7% regression of iris neovascularization (aflibercept). *7–15 mmHg IOP reduction. *23% increased success rate of AGVI when combined preoperatively	Rittiphairoj et al. (202), Yun et al. (2025), Inatani et al. (2021), Hwang and Lee (2021), Guo et al. (2021), Wang et al. (2024b)
Sustained-Release Preparations	Controlled drug release via porous structures or biodegradable carriers with differential pore sizes/degradation rates	*Long-term drug release (3–6 months). *Stable 1OP control (30% reduction in animal models). *Reduced injection frequency	So et al. (2025), Li et al. (2025), Long et al. (2024), Lee et al. (2023), Rodrigo et al. (2021)
Anti-fibrotic/Anti-inflammatory Drugs	*Inhibit trabecular meshwork fibrosis (via TGF-β2, MRTF/SRF pathway). *Reduce intraocular inflammation (via TNF-α, NF-κB pathway)	*50% reduced risk of filtration tract scarring (0.01% epinephrine). *91.7% 12-month patency of filtration tract (5-FU sustained-release). *15.4% additional postoperative IOP reduction (epinephrine in MIGS)	Li et al. (2023), Luo et al. (2022), Chun et al. (2021), He et al. (2024), Masuda et al. (2024), Ichioka et al. (2022)
Neuroprotective Agents	*Protect retinal ganglion cells (via MSC transplantation, AIBP gene therapy). *Regulate neuroinflammation (via HMGB1 inhibition, AMPK/SIRT1 pathway)	*Improved retinal ganglion cell survival (60% increase in animal models with CRISPR-Cas9 + BDNF). *Attenuated optic nerve inflammatory damage	Vilela et al. (2021), Ju et al. (2025), Tonner et al. (2022), Deng et al. (2020)

Abbreviations: VEGF, vascular endothelial growth factor; IOP, intraocular pressure; AGVI, ahmed glaucoma valve implantation; TGF-β2, transforming growth factor beta 2; MRTF/SRF, Myocardin-Related Transcription Factor/Serum Response Factor; TNF-α, tumor necrosis factor alpha; NF-κB, Nuclear Factor Kappa-Light-Chain-Enhancer of Activated B Cells; MIGS, minimally invasive glaucoma surgery; 5-FU, 5-fluorouracil; MSC, mesenchymal stem cell; AIBP, Apolipoprotein A-I binding protein; HMGB1, High Mobility Group Box 1; AMPK/SIRT1, AMP-Activated Protein Kinase/Sirtuin 1; BDNF, Brain-Derived Neurotrophic Factor.

## 3 Clinical application and efficacy: evidence from evidence-based medicine

The aforementioned mechanisms provide a theoretical basis for clinical translation. Below, we validate these therapeutic strategies with evidence from clinical trials, focusing on efficacy, safety, and real-world application scenarios.

### 3.1 Clinical translation of anti-VEGF therapy

In the clinical translation of anti-VEGF therapy, existing studies have confirmed that intravitreal injection of anti-VEGF drugs has a significant effect on NVG. The study shows that a single injection of aflibercept can cause regression of iris neovascularization in 86.7% of NVG patients and reduce the intraocular pressure by an average of 12.3 mmHg (Inatani et al., 2021). Compared with traditional trabeculectomy, anti-VEGF therapy exhibits better safety features. The reported incidence of endophthalmitis is only 0.02%–0.05%, which is significantly lower than the 5%–10% infection risk of filtration surgery. It is worth noting that the combined treatment regimen can further improve the short-term surgical success rate, increasing the intraocular pressure control rate from 68% in simple surgery to 89% (Zhou et al., 2023; He et al., 2024).

### 3.2 Efficacy verification of sustained-release technology​

Regarding the application progress of sustained-release technology, current research focuses on solving the problem of frequent injections. Experimental sustained-release systems have shown that a single intravitreal implantation of intraocular pressure-lowering drugs can maintain effective concentrations for up to 6 months and can continuously reduce intraocular pressure by 30% in animal models (Rodrigo et al., 2021; Shen et al., 2023). Of particular note is that sustained-release preparations loaded with 5-fluorouracil (5-FU) using nanocarrier technology have demonstrated significant anti-fibrotic effects in applications after glaucoma filtration surgery, increasing the 12-month patency rate of the filtration tract to 91.7%, which is 23% higher than that of traditional intermittent injection regimens (Wang X. et al., 2024; Patel and Sheth, 2021). These technological breakthroughs provide new ideas for reducing injection frequency and improving patient compliance (Masuda et al., 2024).

### 3.3 Application scenarios of intraoperative adjuvant Injection​

In terms of combined treatment strategies involving intraoperative adjuvant injection, clinical evidence supports the synergistic effect of MIGS combined with drug therapy. Studies have shown that intraoperative anterior chamber angle injection of epinephrine during iStent implantation can reduce Schlemm’s canal collapse through α1 receptor agonism, resulting in an additional 15.4% reduction in postoperative intraocular pressure (p < 0.01) (Ichioka et al., 2022; Jain and Fan Gaskin, 2025). For high-risk NVG cases, anti-VEGF injection 72 h before surgery can increase the success rate of AGVI from 62% to 84% and reduce intraoperative bleeding (Fan et al., 2023; Sahin et al., 2024). In terms of anti-scarring management, the modified 5-FU sustained-release technology reduces the failure rate of filtration surgery from 38% with conventional treatment to 8% by inducing fibroblast apoptosis, without increasing corneal endothelial cell loss (Wang X. et al., 2024; Fang et al., 2022). These combined regimens are reshaping the treatment paradigm for refractory glaucoma (Siedlecki et al., 2022; Burgos-Blasco et al., 2022).

## 4 Challenges and future directions

While clinical data confirm the value of intravitreal therapy, several limitations hinder its widespread adoption. This section addresses current challenges and explores breakthrough technologies to overcome these barriers.

### 4.1 Existing problems

The core challenge of intravitreal injection in glaucoma treatment lies in the limitations of drug delivery systems. Although current anti-VEGF therapy can effectively control the progression of neovascular glaucoma, its efficacy is highly dependent on the control of primary diseases. Literature shows that the recurrence rate of diabetic retinopathy patients after treatment still exceeds 30% (Zhou et al., 2023). Sustained-release implants can prolong the drug action time, but there is a 5%–8% risk of displacement, and there is a lack of means for real-time monitoring of drug concentrations in aqueous humor (Rodrigo et al., 2021; Hughes et al., 2023). More critically, the relationship between dynamic changes of aqueous humor cytokines and drug responsiveness remains unclear. For example, the concentration of TGF-β2 in aqueous humor of patients with primary open-angle glaucoma is significantly increased, being 2-3 times higher than that in normal individuals, but it is downregulated in secondary open-angle glaucoma (Igarashi et al., 2021; Fujimoto et al., 2023). This complex regulatory network makes the formulation of individualized drug delivery schemes face significant technical bottlenecks (Wu et al., 2025).

### 4.2 Outlook on breakthrough technologies​

The development of breakthrough technologies is focusing on intelligent, responsive delivery systems. Thermosensitive hydrogels can achieve intraocular pressure threshold-responsive drug release through temperature-sensitive polymers, such as chitosan-amino acid complexes. *In vitro* experiments have confirmed that they can maintain sustained drug release for more than 28 days (Luo et al., 2023; Tong et al., 2022). Nanorobot technology has demonstrated the ability to target and clear fibrotic deposits in the trabecular meshwork in mouse models, and has been shown to reduce intraocular pressure by 35% by activating the Piezo Type Mechanosensitive Ion Channel Component 1 (Piezo1) mechanosensitive channel (Morozumi et al., 2022; Wang et al., 2025). A more cutting-edge Clustered Regularly Interspaced Short Palindromic Repeats and CRISPR-associated protein 9 (CRISPR-Cas9) gene editing combined therapy is being explored. CRISPR-Cas9 works like a “molecular scissors—it can precisely “cut” abnormal genes (such as those overproducing VEGF) and replace them with healthy ones, while also delivering neuroprotective factors to save damaged retinal cells. Through nanocarrier delivery systems, it can achieve the synergistic release of VEGF gene knockout and neurotrophic factors such as Brain-Derived Neurotrophic Factor (BDNF). Animal experiments show that this strategy can increase the survival rate of retinal ganglion cells by 60% (Singh et al., 2025; Deng et al., 2020). These innovative technologies provide new ideas for overcoming the limitations of traditional drug delivery methods (Shen et al., 2023; Corelli, 2021).

### 4.3 Optimization path for clinical practice​

Multi-omics technologies are reshaping the paradigm of precise glaucoma treatment. The latest research has found that dynamic changes in aqueous humor biomarker profiles, including IL-6, BDNF, and Transforming Growth Factor Beta (TGF-β), are significantly associated with drug sensitivity. For example, IL-6 trans-signaling can inhibit TGF-β-mediated trabecular meshwork fibrosis (Igarashi et al., 2021; Urahashi et al., 2025). By integrating genomic, proteomic, and metabolomic data, researchers have identified a direct link between mutations in Wingless-type (Wnt) ligand secretion mediator genes and impaired function of the trabecular meshwork (the eye’s key “drainage filter”) (Castillo-Plata et al., 2022). For example, patient-specific trabecular meshwork models—built using induced pluripotent stem cell (iPSC) technology and combined with microfluidic chip systems—act like “miniature eye labs,” allowing doctors to test how a patient might respond to different drugs in a dish before actual treatment (Tian et al., 2020). These tools lay the groundwork for a personalized “treatment-monitoring-adjustment” loop: first, use genetic and molecular data to select the right therapy; then, track biomarkers (such as aqueous humor cytokines) to monitor effectiveness; and finally, adjust the treatment plan based on real-time feedback (Jiang et al., 2025). The optimization of clinical practice requires comprehensive consideration of technological innovation and translational medicine. Existing evidence indicates that anti-VEGF drugs combined with surgery can increase the short-term success rate of neovascular glaucoma patients (Zhou et al., 2023), but attention should still be paid to intraocular pressure fluctuations caused by long-term repeated injections (de Vries et al., 2020; Vilares-Morgado et al., 2023). Future development directions include developing aqueous humor instant detection devices based on quantum dot sensors (Wu et al., 2025), designing degradable drug delivery systems (Kompella et al., 2021), and using artificial intelligence algorithms to integrate multi-modal data for predicting treatment responses (Egger et al., 2025). It is worth noting that interdisciplinary technologies such as self-generating electricity systems have been confirmed in mouse models to achieve automatic intraocular pressure regulation through trabecular meshwork contraction (Wang et al., 2025), which indicates that glaucoma treatment is rapidly moving towards intellectualization, mini-invasiveness, and precision.

## 5 Discussion

Integrating mechanistic insights, clinical evidence, and technological prospects, we now synthesize the transformative impact of intravitreal injection on glaucoma management and outline its future direction.

I. Intravitreal injection has emerged as a new paradigm in glaucoma treatment by breaking through the blood-ocular barrier and precisely regulating the neurovascular microenvironment with anti-VEGF drugs, sustained-release preparations, and other agents. Its combination with surgery can improve success rates and reduce complications, while technologies such as nanocarriers have addressed the limitations of traditional drug delivery.
II. Current challenges include drug efficacy being dependent on the control of primary diseases and the risk of carrier displacement.
III. Future efforts need to overcome the bottlenecks in carrier intelligence and individualized delivery, and rely on multidisciplinary collaboration involving gene technology, minimally invasive surgery, and real-time monitoring to achieve a leap from “intraocular pressure reduction” to “visual function repair”. Perhaps the keyword that will subvert glaucoma surgery in the future will no longer be “minimally invasive”; each glaucoma patient may only need to receive regular intraocular injections of corresponding drug preparations to achieve good intraocular pressure control and optic nerve protection.

## Glossary

Aqueous humor: The clear fluid that fills the front part of the eye, maintaining eye pressure
Anti-VEGF: Drugs that inhibit vascular endothelial growth factor (VEGF), a protein that stimulates abnormal blood vessel formation
Intraocular pressure (IOP): The pressure inside the eye; high IOP is a major risk factor for glaucoma
Intravitreal injection: Direct injection of drugs into the vitreous humor (the gel-like substance in the eye’s center) to target retinal or intraocular tissues
MIGS (Minimally Invasive Glaucoma Surgery): Small-scale surgical procedures that enhance fluid drainage from the eye, with lower complication rates than traditional surgery
Neovascular glaucoma (NVG): A severe form of glaucoma caused by abnormal blood vessel growth in the eye, often linked to retinal diseases like diabetes
Sustained-release preparations: Drug delivery systems that release medications gradually over time, reducing injection frequency
Trabecular meshwork: A sponge-like structure in the eye that drains fluid; damage here can raise eye pressure
